# Mouse lymph node colonization models predict human tumor metastasis in melanoma

**DOI:** 10.64898/2026.09.22.753675

**Published:** 2026-09-23

**Authors:** Brendan K. Ball, Reem Al-Humadi, Sabrina Zdravkovic, Ilayda Ilerten, Brooks A. Benard, Brooke E. Howitt, Andrew J. Gentles

## Abstract

Aggressive tumor progression and distant metastases of cancers such as cutaneous melanoma are thought to be preceded by regional lymph node (LN) infiltration. Previous mouse studies have selectively enriched for tumor cells with enhanced LN migratory capacity but the mechanistic biology underpinning this proclivity has not been directly linked to human outcomes. Likewise, the exact role LN colonization plays in distant metastasis in humans remains unclear. To translate and conserve findings from mouse models to humans, we used a computational framework termed Translatable Components Regression (TransComp-R) to integrate mouse LN tumor samples with human primary and metastatic tumors. We identified transcriptional programs of early- and late-stage mouse tumor generations that stratified primary and metastatic tumors from patients with melanoma. These transcriptional programs associated with immune function, cell cycle checkpoints, and DNA-to-protein biosynthesis pathways. We then employed EcoTyper and identified six distinct melanoma-specific cellular communities (ecotypes) comprising co-occurring cell states. Of these six melanoma ecotypes, we identified a community strongly associated with primary tumor cells, while the other five represented a spectrum of metastatic tumor microenvironments with increased adverse outcomes. These ecotypes and their significantly expressed genes may contribute to the tumor-immune microenvironment that facilitates LN colonization in melanoma. We further validated the physical co-localization of these melanoma ecotypes using spatial transcriptomics. Our integration of pre-clinical models with human data reveals potential biological pathways and transcriptomic signatures for future investigation and supports the utility of mouse models in studying cancer metastasis.

**GRAPHICAL ABSTRACT:**

## Full Text Availability

The license terms selected by the author(s) for this preprint version do not permit archiving in PMC. The full text is available from the preprint server.

